# Supplementary material for: 20th century cooling of the deep ocean contributed to delayed acceleration of Earth’s energy imbalance
Source: Nat Commun. 2021 Jul 29;12:4604. doi: 10.1038/s41467-021-24472-3 (PMC8322321; doi:10.1038/s41467-021-24472-3)
Supplement: Supplementary file 1 — Supplementary Materials [file 41467_2021_24472_MOESM1_ESM.pdf]

nature


# Supplementary Materials: 20th Century Cooling of the Deep Ocean Contributed to Delayed Acceleration of Earth's Energy Imbalance

Aaron Bagnell and Timothy DeVries

## Validating the mapping method

One of the major challenges faced by any mapping method for estimating ocean heat content (OHC) changes are the presence of sampling biases and significant geophysical noise in the observed dataset. Sampling biases arise from the sparse and irregular sampling of ocean temperature observations. Due to the relative difficulty of retrieving observations from remote regions of the ocean, sampling has historically favored coasts, the Northern Hemisphere, and depths shallower than 700 m, leaving much of the ocean unobserved. Given that temperature sampling in the historical record is also heavily biased towards the Argo era (the period after 2005), our method seeks to leverage the autocorrelation of temperature anomalies by using the well sampled near-surface and present periods to predict the anomaly fields for the ocean interior at depths greater than 2000 m and for years prior to 2005 when ocean temperature data is sparser.

To demonstrate that our mapping method is robust to irregular sampling coverage, we used the sparsity of the combined instrumental datasets (see Methods) to decimate simulated temperature fields from individual historical runs of the MIROC (r1i1p1f1)[19] and CNRM (r1i1p1f2)[20] CMIP6 models, interpolated to the WOA grid. These were obtained from the CMIP6 data archive at https://esgf-node.llnl.gov/projects/cmip6/, last accessed on Jul. 16, 2020. The decimated model temperature fields then have the same data sparsity as the observations on the WOA grid. We generate a monthly climatology by averaging the model data in the period 2005-2014, which we then subtract from the temperature fields to create the temperature anomalies. Then we apply the same 12-month moving average filter and bin the data to 6-month time-steps, as we do with the observations. We next use the ARANN procedure (see Methods and Supplementary Fig. 1) to interpolate the decimated model data in an attempt to recreate the original model temperature anomalies. For our test, we ran the ARANN gap-filling backwards from July 2014 to January 1946, then forwards again across the same time interval, producing 30 ensemble members for each CMIP6 model. We found that the OHC calculated from the ARANN-reconstructed temperature anomalies matched the original modeled CMIP6 OHC very well on global and basin scales (Supplementary Figs. 4-7) with minimal bias that generally did not exceed the uncertainty (2 standard deviations) of our mapping method.

The ability of the ARANN procedure to accurately reconstruct the modeled OHC changes from observed sparsity is reassuring and demonstrates that the ARANN is robust to the presence of large observational gaps. However, the task of accurately reconstructing the modeled temperature anomalies is easier than reconstructing the observed temperature anomalies, since sub-mesoscale processes in the ocean produce additional variability in the temperature observations that is not present in the coarse-resolution models. Comparing the residual error between the ARANN reconstruction and the modeled temperature anomalies (Supplementary Figs. 8-11, d-e), and the corresponding residual error between the ARANN reconstruction and the observed temperature anomalies (Supplementary Fig. 3, c, f, i, l), it is clear that the residuals are far larger and more randomly distributed for the difference between the ARANN and

observations, than for the difference between the ARANN and CMIP6 models. This is also demonstrated in Supplementary Fig. 12 for the residuals between the ARANN reconstruction and the CMIP6 models and observations over time in both the shallow (0-700 m) and deep (700-5500 m) ocean.

To assess the ability of the ARANN to reconstruct the "true" temperature variability in the presence of small-scale variability that affects real-world observations, we added simulated geophysical noise fields to the CMIP6 modeled temperature anomalies. For these noise fields, we used the residuals between a single member of the ensemble of ARANN-reconstructed temperature anomalies and observed temperature anomalies at each time-step and depth level, for example as shown in Supplementary Fig. 3 (c, f, i, l) for a couple times and depth levels. We interpret these residuals as due primarily to the geophysical noise present in the observations, although it also includes any additional systematic biases in our interpolation versus the observations. After adding these residuals to the CMIP6 modeled temperature anomaly fields, we then repeated our data processing and interpolation procedure to see if the ARANN could still faithfully reproduce the original OHC trends in the presence of this more realistic temperature variability.

When interpolating the CMIP6 temperature anomalies with this additional noise that mimics the geophysical noise in the observations, the residuals between the ARANN-reconstructed and the modeled temperature anomalies are larger and more randomly distributed (Supplementary Figs. 8-11, g) than those reconstructed from the original modeled temperature anomalies (Supplementary Figs. 8-11,f). The effect of geophysical noise on the ARANN reconstruction is also shown in Supplementary Fig. 12 for the residuals over time: by adding realistic geophysical noise to the CMIP6 models, we obtain residual errors that are similar in magnitude to those obtained from the observations in both the shallow (0-700 m) and deep (700-5500 m) oceans, indicating that small-scale variability has been adequately accounted for in our validation. Even with the larger residuals, the underlying large-scale temperature anomaly patterns are reconstructed well (Supplementary Figs. 8-11, compare panels d and e to a). The residuals show features on the order of ~$10^3$ km (Supplementary Figs. 8-11, f-g), which is smaller than what is needed to assess global and basin-scale OHC. The reconstructions are also robust to regions with large amounts of missing data. For example, at 1000 m in the year 1960 (Supplementary Fig. 10), sampling biases have left the South Pacific and Southern Ocean with large gaps in coverage. Nevertheless, patterns in these regions are reconstructed with reasonable accuracy.

Importantly for the results discussed in the main text, the bias on the global and basin scale remains relatively small and almost always of lesser magnitude than the uncertainty (Supplementary Figs. 4-7), although the uncertainty roughly doubles when geophysical noise is included. The performance does somewhat depend on the distribution of temperature anomalies, which is governed by processes in the underlying model. For instance, the ARANN reconstruction (both with and without added geophysical noise) for the CNRM OHC contains a systematic bias that slightly exceeds the estimated uncertainty for the period prior to 1955 and depths greater than 2000 m (Supplementary Fig. 5). This is not the case with the MIROC reconstructions (Supplementary Fig. 4), indicating that the impact of sampling bias will depend on the "true" anomaly field.

Further comparing the temperature anomaly fields of the original MIROC CMIP6 model run versus a single realization of the ARANN reconstruction (Supplementary Figs. 8-11), we find that large scale patterns can be well reproduced both with and without added geophysical noise.

For the upper 50 m in the year 1960 (Supplementary Fig. 8), the ARANN recreates most large-scale patterns, though the presence of geophysical noise yields somewhat larger residuals (Supplementary Fig. 8f vs. 8g). The impact of geophysical noise becomes even more apparent in the year 2010, where in the upper 50 m there is very even sampling (Supplementary Fig. 9). Here the residual temperature anomalies are smaller than in 1960, but the addition of geophysical noise still leads to anomalies that are both larger in magnitude and spatial extent. In 1960 at 900-1100 m (Supplementary Fig. 10), sampling coverage is even sparser than in the upper 50 m, but the ARANN reconstruction still performs well at reconstructing the original temperature anomaly fields ($R^2$ = 0.69), although the addition of geophysical noise reduces the performance substantially ($R^2$ = 0.43) in the presence of such sparse data. In 2010, there is much better sampling coverage in the deep ocean (Supplementary Fig. 11), but the ARANN performance is similar to that in 1960, with an $R^2$ of 0.67 without geophysical noise, and 0.31 with geophysical noise. At all times and depths, the residuals between the ARANN reconstruction and the original modeled temperature anomalies remain relatively small-scale (<~1000 km), even in the presence of realistic geophysical noise (Supplementary Figs. 8-11, f-g). We therefore conclude that the ARANN can overcome both biases in sampling coverage and geophysical noise to reproduce most regional and basin-scale features necessary to assess changes in OHC over time.

**Supplementary Fig. 1. Overview of the temperature data processing steps**

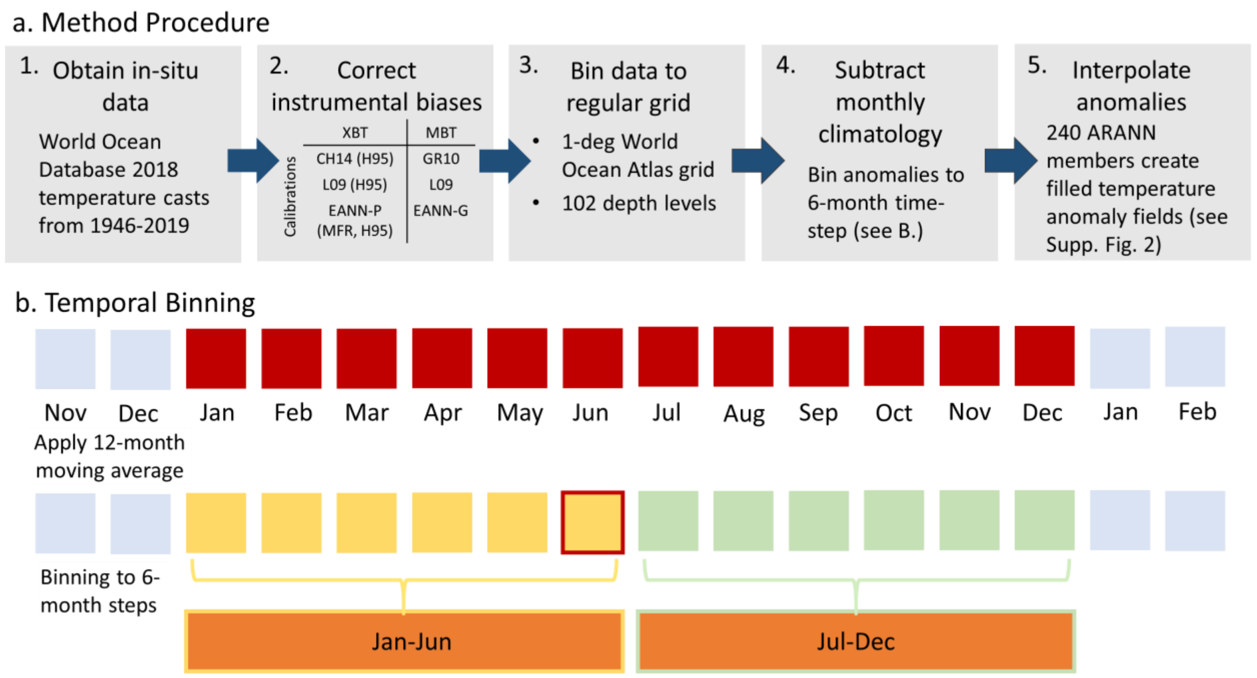


(a) Summary of the data processing steps for creating temperature anomaly fields from raw temperature observations, and (b) expansion of Step 4 in (a) demonstrating the procedure for smoothing and binning these temperature anomalies to a 6-month time-step.

**Supplementary Fig. 2. Description of the ARANN interpolation method**

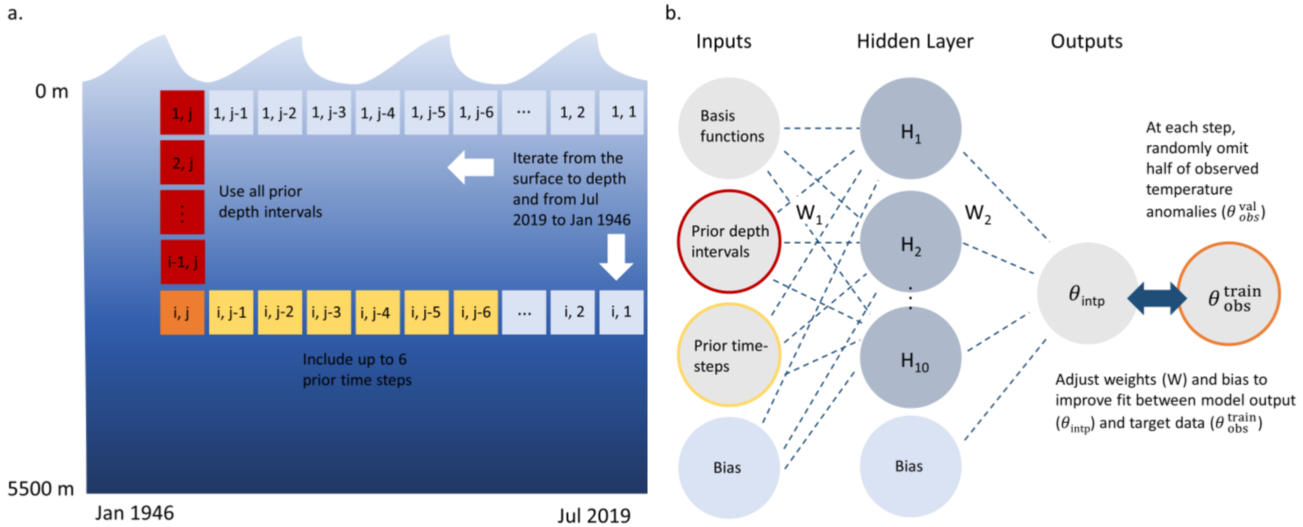


115 Schematic of (a) the iterative process of the ARANN method, which propagates information from near the sea surface and in modern times to less sampled time periods and depths, and (b) the architecture of the ARANN, which uses sinusoidal basis functions, prior time-steps, and prior depth levels to estimate temperature anomalies at the current time-step.

**Supplementary Fig. 3. Observed and interpolated temperature anomalies**

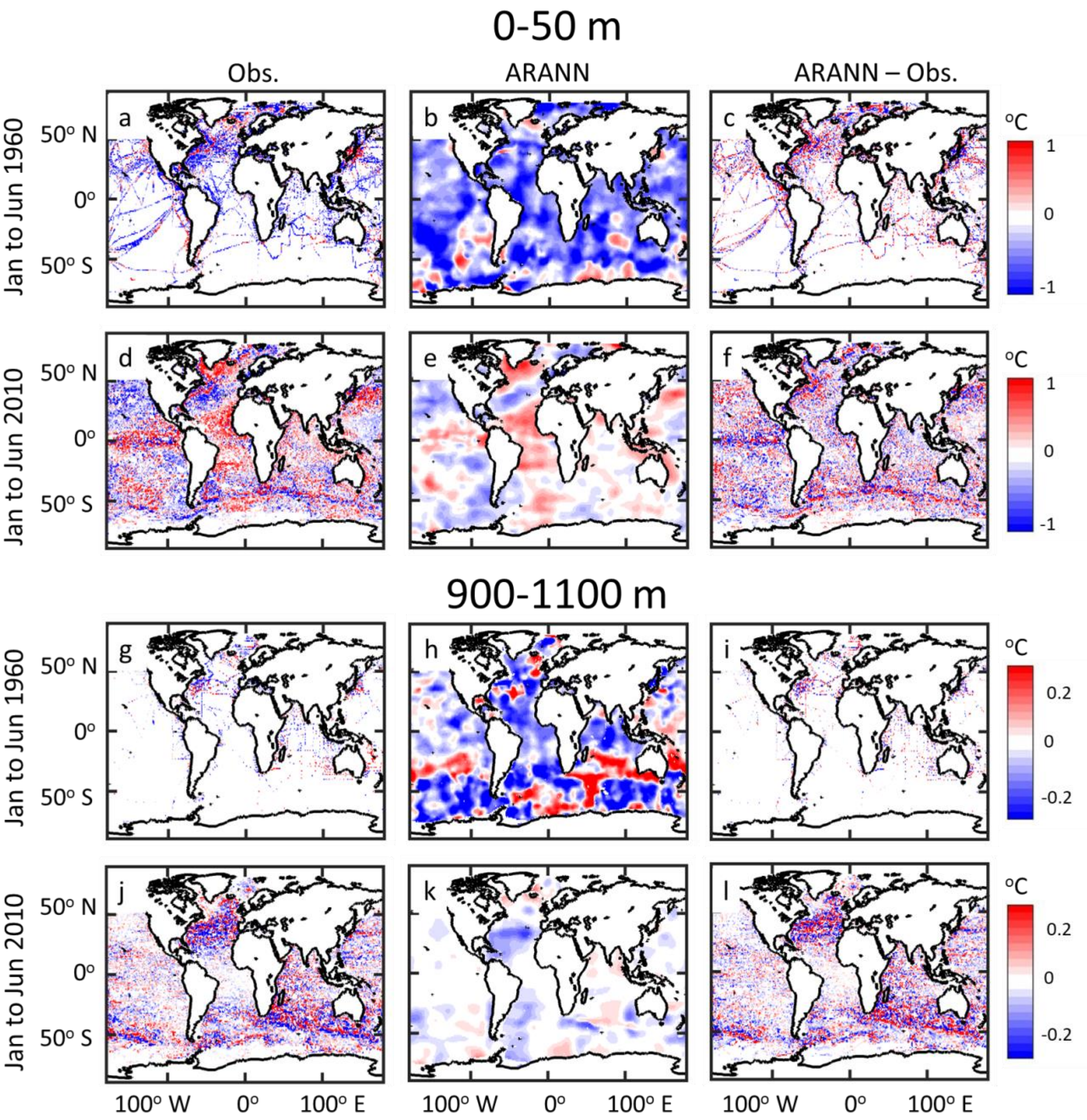


(Top panels) Temperature anomalies for the 0-50 m depth interval for (a-c) January to June 1960 and (d-e) January to June 2010 from (a,d) observations, (b,e) a single realization of the ARANN interpolation of the observed field, and (c,f) the residuals between the ARANN product and the original observations. (Bottom panels) Temperature anomalies for the 900-1100 m depth interval for (g-i) January to June 1960 and (j-l) January to June 2010 from (g,j) observations, (h,k) a single realization of the ARANN interpolation of the observed field, and (i,l) the residuals between the ARANN product and the original observations.

**Supplementary Fig. 4. Global ocean heat content reconstructions of the MIROC CMIP6 model**

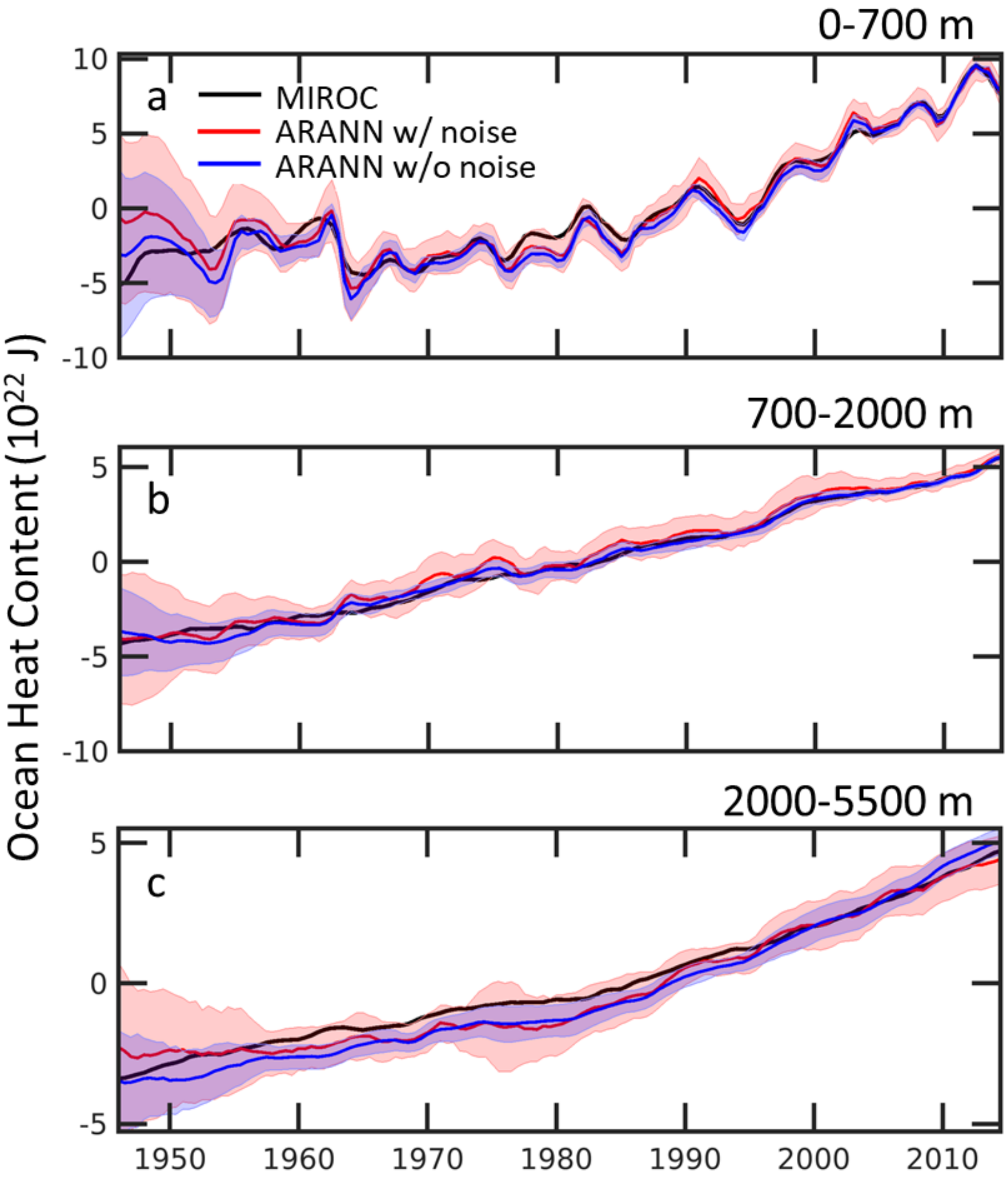


Global ocean heat content estimates based on an original MIROC CMIP6 climate model simulation (black), the ARANN reconstruction based on modeled temperature anomalies reduced to observational sparsity (ARANN w/o noise, blue), and the ARANN reconstruction based on modeled temperature anomalies with additional noise added to mimic geophysical noise occurring in the observations (ARANN w/ noise, red) for the depth intervals (a) 0-700 m, (b) 700-2000 m, and (c) 2000-5500 m. Error bars for the ARANN reconstructions are the 2 standard deviation range across 30 ensemble members.

**Supplementary Fig. 5. Global ocean heat content reconstructions of the CNRM CMIP6 model**

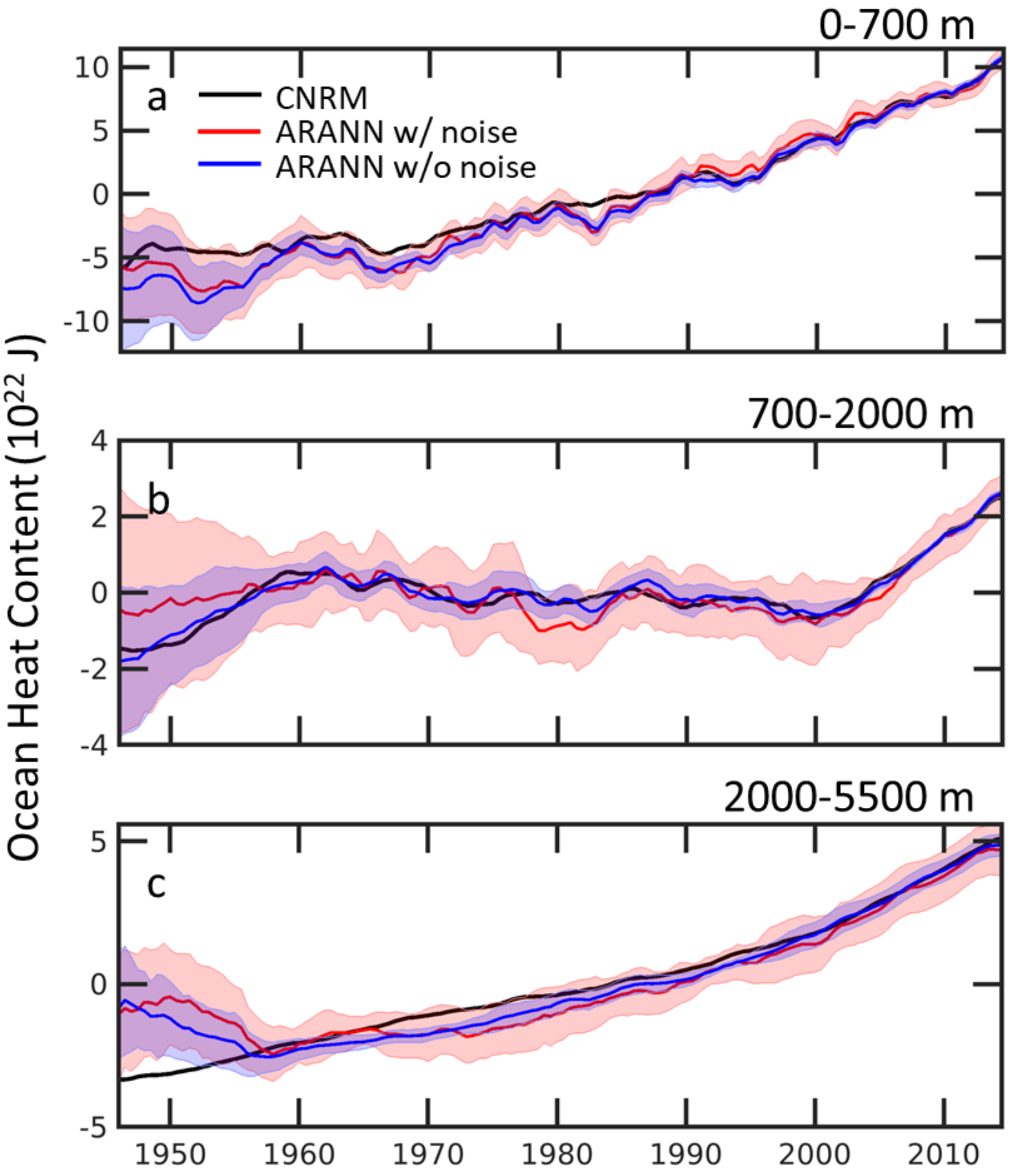


Global ocean heat content estimates based on an original CNRM CMIP6 climate model simulation (black), the ARANN reconstruction based on modeled temperature anomalies reduced to observational sparsity (ARANN w/o noise, blue), and the ARANN reconstruction based on modeled temperature anomalies with additional noise added to mimic geophysical noise occurring in the observations (ARANN w/ noise, red) for the depth intervals (a) 0-700 m, (b) 700-2000 m, and (c) 2000-5500 m. Error bars for the ARANN reconstructions are the 2 standard deviation range across 30 ensemble members.

**Supplementary Fig. 6. Ocean basin heat content reconstructions of the MIROC CMIP6 model**

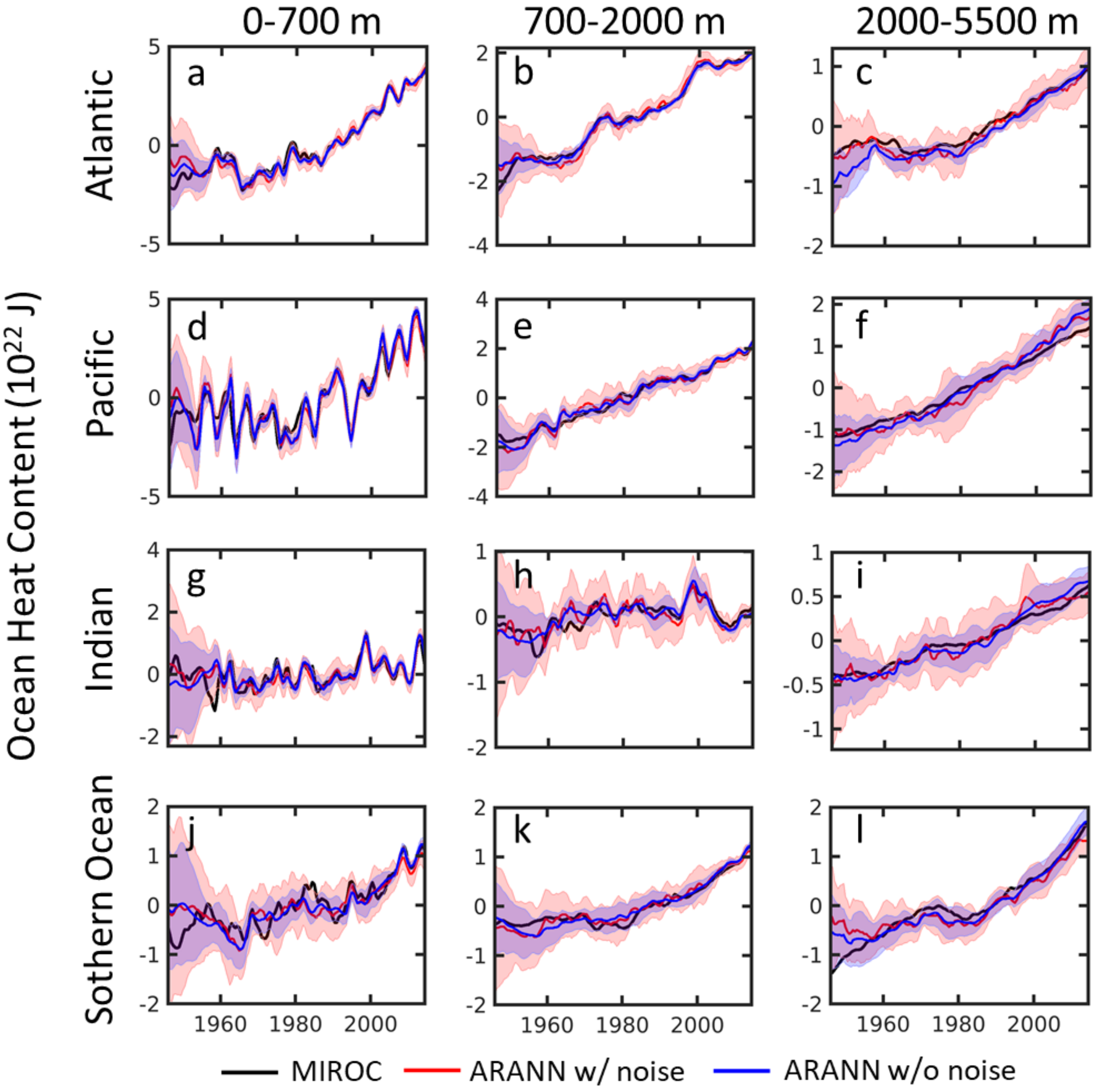


Basin-scale ocean heat content (OHC) anomalies based on an original MIROC CMIP6 climate model run (black), the ARANN reconstruction based on modeled temperature anomalies reduced to observational sparsity (ARANN w/o noise, blue), and the ARANN reconstruction based on modeled temperature anomalies with additional noise added to mimic geophysical noise occurring in the observations (ARANN w/ noise, red). OHC is presented for the Atlantic Ocean at depth intervals (a) 0-700 m, (b) 700-2000 m, (c) 2000-5500 m; for the Pacific Ocean at depth intervals (d) 0-700 m, (e) 700-2000 m, (f) 2000-5500 m; for the Indian Ocean at depth intervals (g) 0-700 m, (h) 700-2000 m, (i) 2000-5500 m; and for the Southern Ocean at depth intervals (j) 0-700 m, (k) 700-2000 m, (l) 2000-5500m. Error bars for the ARANN reconstructions are the 2

standard deviation range across 30 ensemble members. Ocean basins are defined using the World Ocean Atlas mask[58], with the Southern Ocean considered everything south of $50^{o}$ S.

**Supplementary Fig. 7. Ocean basin heat content reconstructions of the CNRM CMIP6 model**

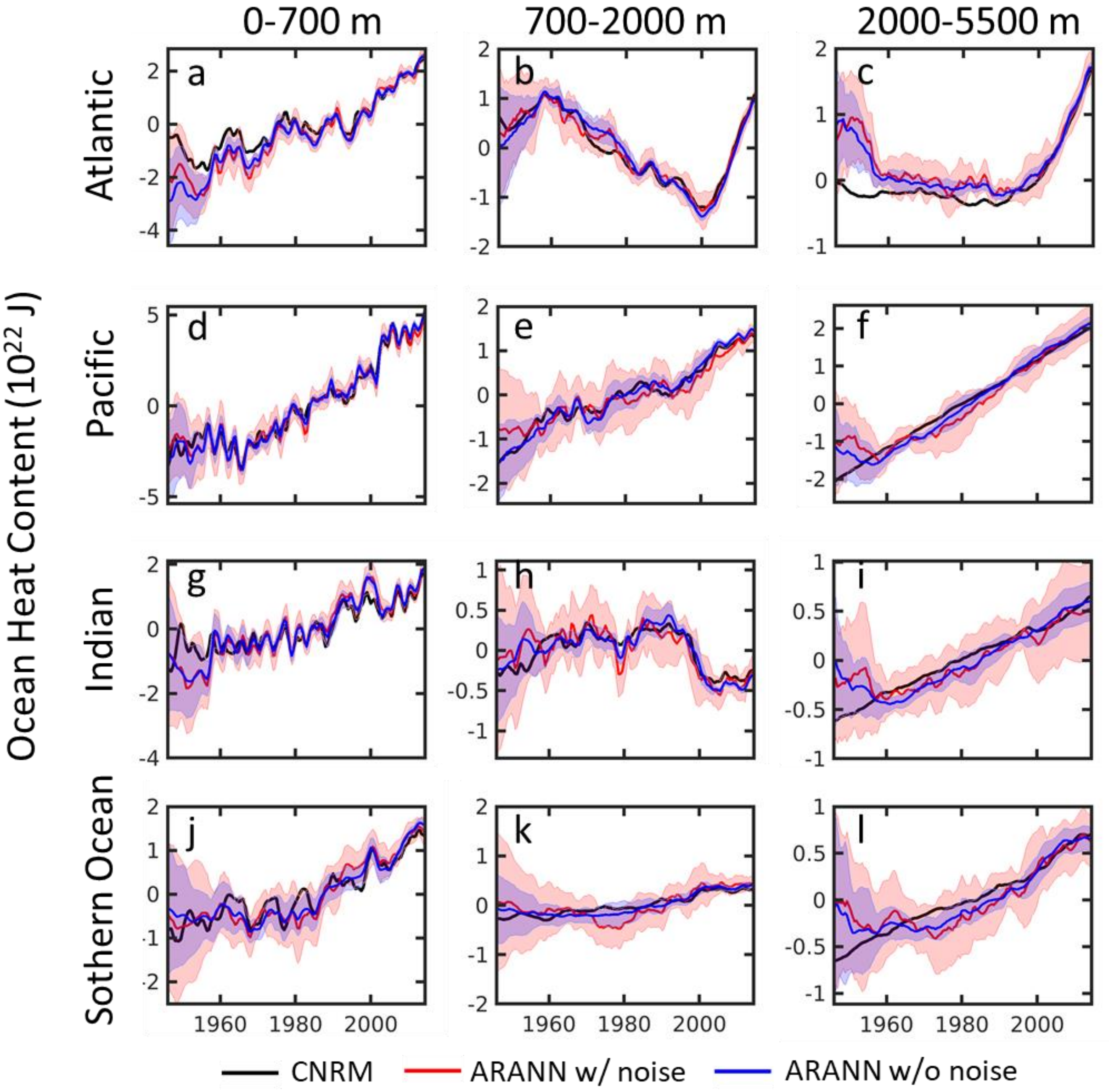


Basin-scale ocean heat content (OHC) anomalies based on an original CNRM CMIP6 climate model run (black), the ARANN reconstruction based on modeled temperature anomalies decimated to observational sparsity (ARANN w/o noise, blue), and the ARANN reconstruction based on modeled temperature anomalies with additional noise added to mimic geophysical noise occurring in the observations (ARANN w/ noise, red). Panels and definitions are the same as those defined for Supplementary Figure 6.

**Supplementary Fig. 8. Near-surface maps of the ARANN reconstruction for the year 1960**

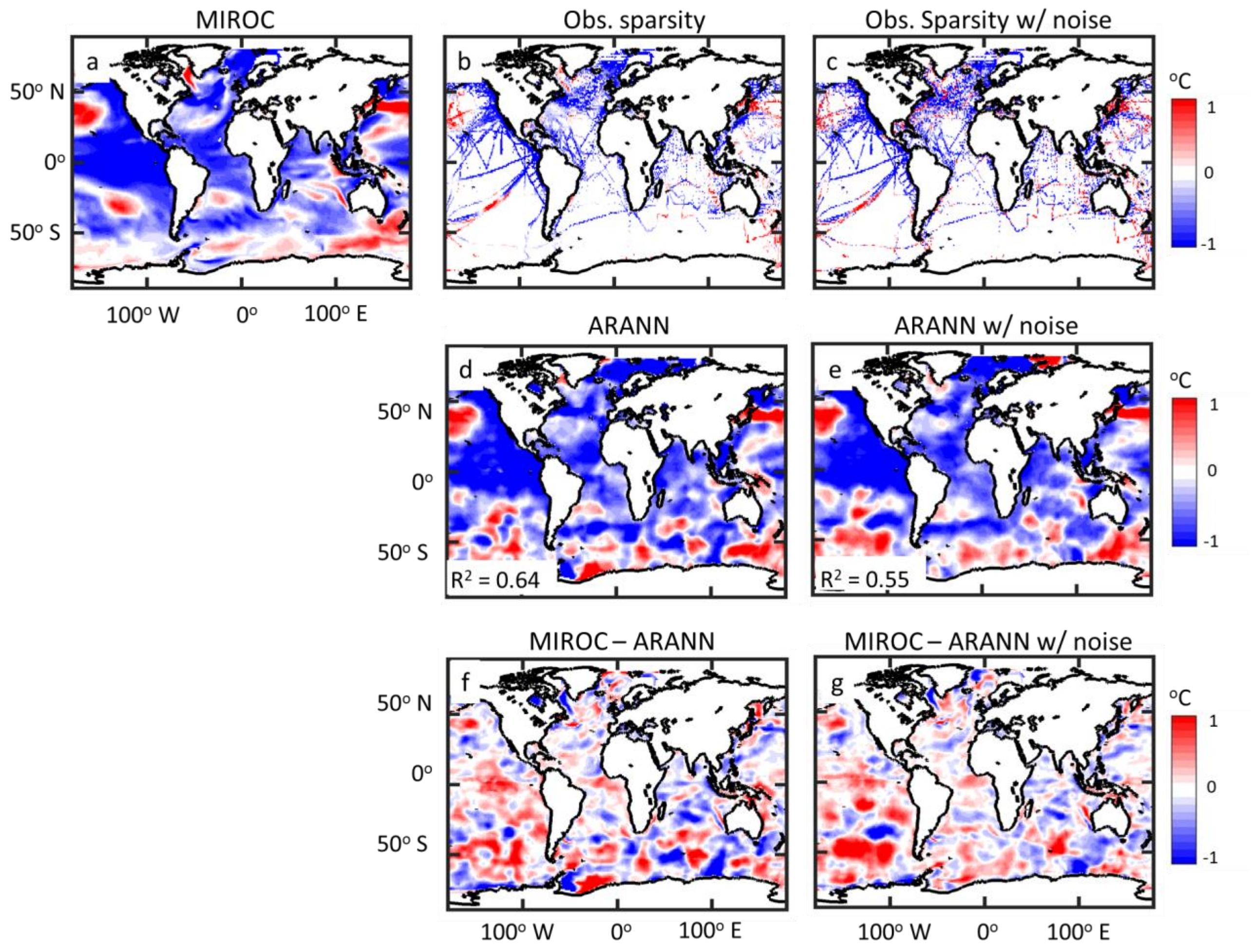


Temperature anomalies for Jan-Jun 1960 over the 0-50 m depth interval for (a) a MIROC CMIP6 model run, (b) the MIROC model after being reduced to observed sparsity, and (c) the MIROC model at observed sparsity with added geophysical noise. The middle row shows (d) a single realization of the ARANN interpolation of the MIROC temperature anomalies and (e) the ARANN interpolation of the MIROC temperature anomalies with added geophysical noise. The bottom row shows (f) residuals between the original MIROC model and the ARANN interpolation and (g) residuals using an ARANN interpolation with additional geophysical noise added to the modeled temperature anomalies. The middle panels also show the $R^2$ between ARANN-reconstructed temperature anomalies (shown in each respective panel) and the original MIROC temperature anomalies in panel (a).

**Supplementary Fig. 9. Near-surface maps of the ARANN reconstruction for the year 2010**

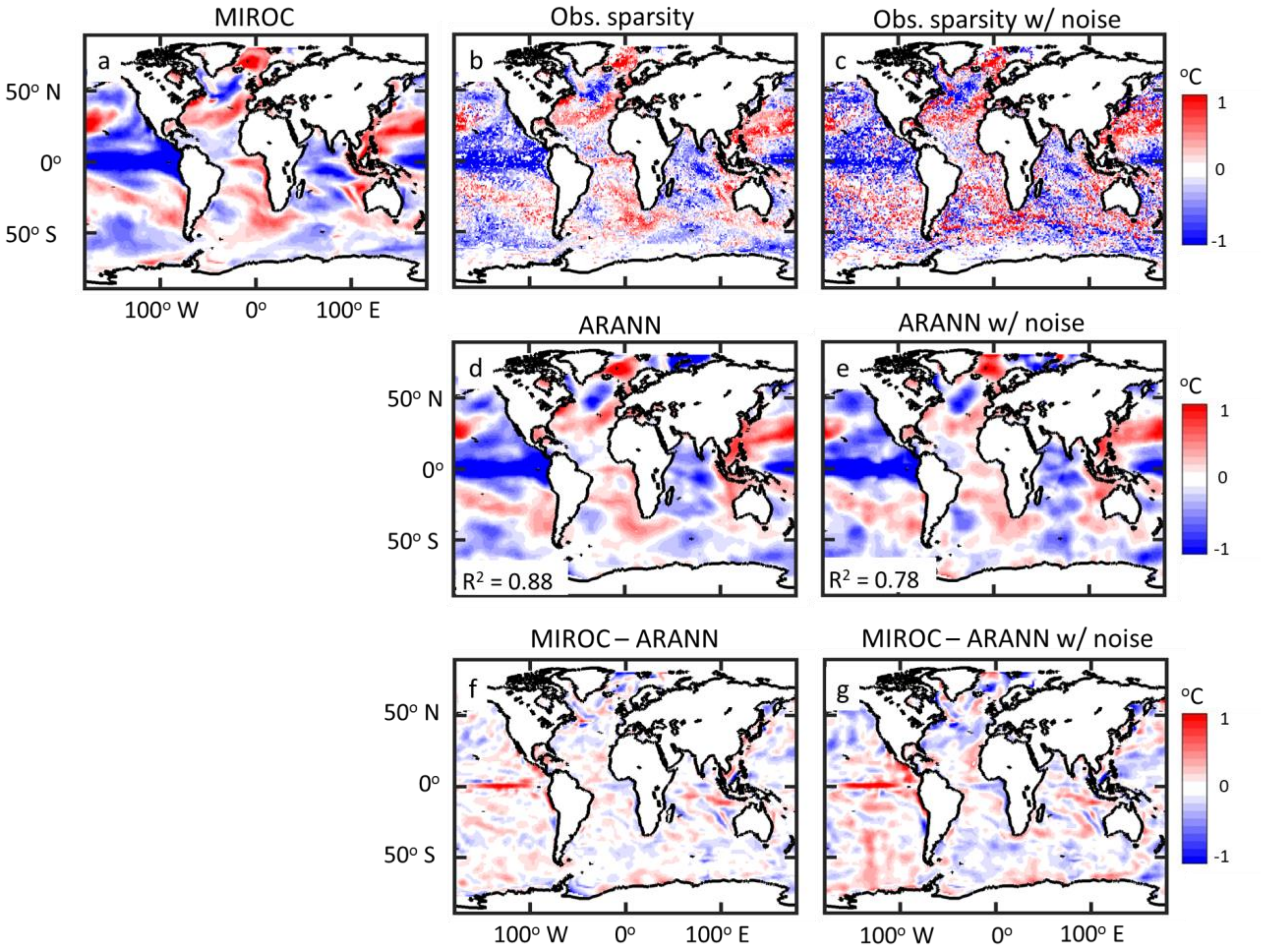


Temperature anomalies for Jan-Jun 2010 over the 0-50 m depth interval for (a) a MIROC CMIP6 model run, (b) the MIROC model after being reduced to observed sparsity, and (c) the MIROC model at observed sparsity with added geophysical noise. The middle row shows (d) a single realization of the ARANN interpolation of the MIROC temperature anomalies and (e) the ARANN interpolation of the MIROC temperature anomalies with added geophysical noise. The bottom row shows (f) residuals between the original MIROC model and the ARANN interpolation and (g) residuals using an ARANN interpolation with additional geophysical noise added to the modeled temperature anomalies. The middle panels also show the $R^2$ between ARANN-reconstructed temperature anomalies (shown in each respective panel) and the original MIROC temperature anomalies in panel (a).

**Supplementary Fig. 10. 1000m maps of the ARANN reconstruction for the year 1960**

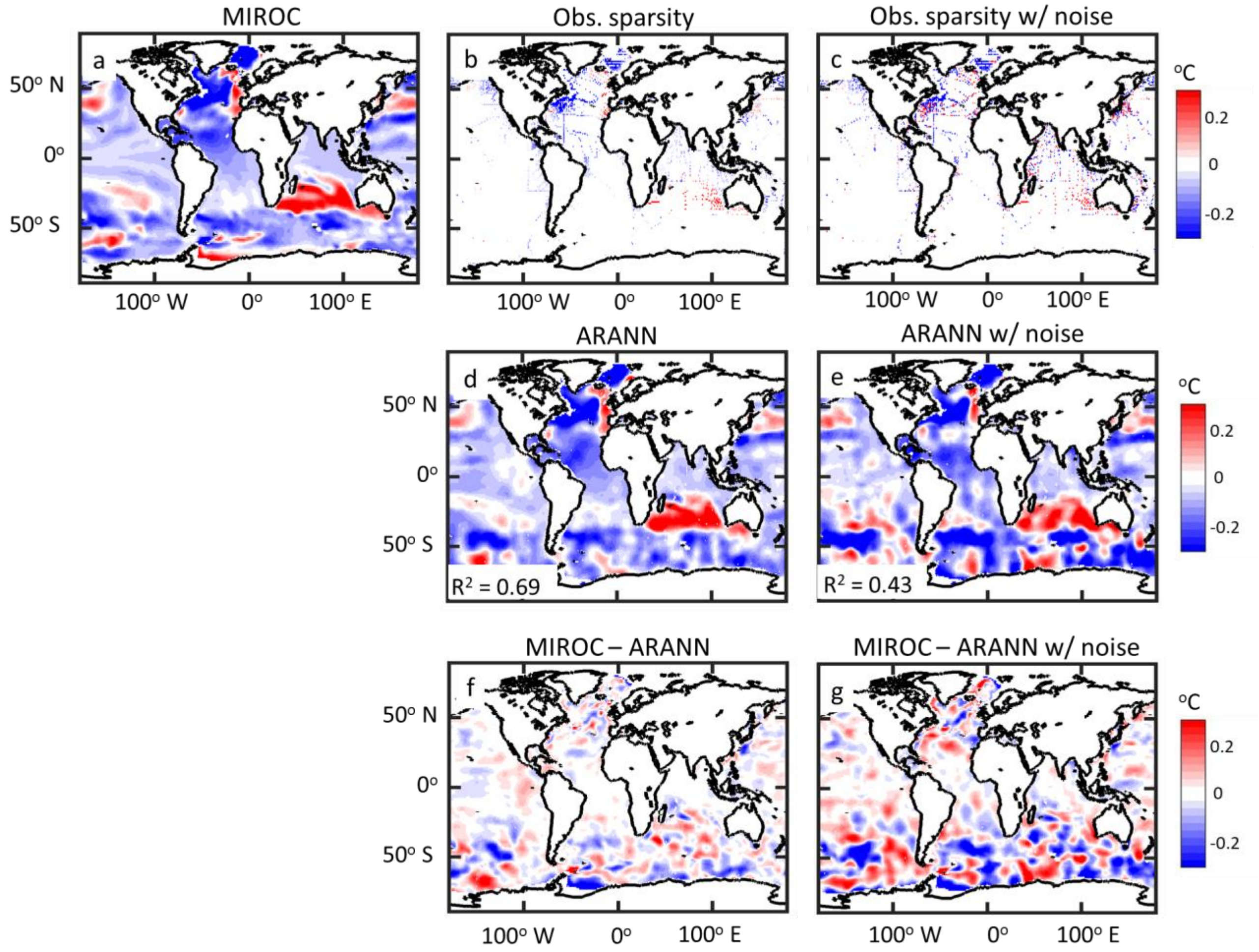


Temperature anomalies for Jan-Jun 1960 over the 900-1100 m depth interval for (a) a MIROC CMIP6 model run, (b) the MIROC model after being reduced to observed sparsity, and (c) the MIROC model at observed sparsity with added geophysical noise. The middle row shows (d) a single realization of the ARANN interpolation of the MIROC temperature anomalies and (e) the ARANN interpolation of the MIROC temperature anomalies with added geophysical noise. The bottom row shows (f) residuals between the original MIROC model and the ARANN interpolation and (g) residuals using an ARANN interpolation with additional geophysical noise added to the modeled temperature anomalies. The middle panels also show the $R^2$ between ARANN-reconstructed temperature anomalies (shown in each respective panel) and the original MIROC temperature anomalies in panel (a).

**Supplementary Fig. 11. 1000m maps of the ARANN reconstruction for the year 2010**

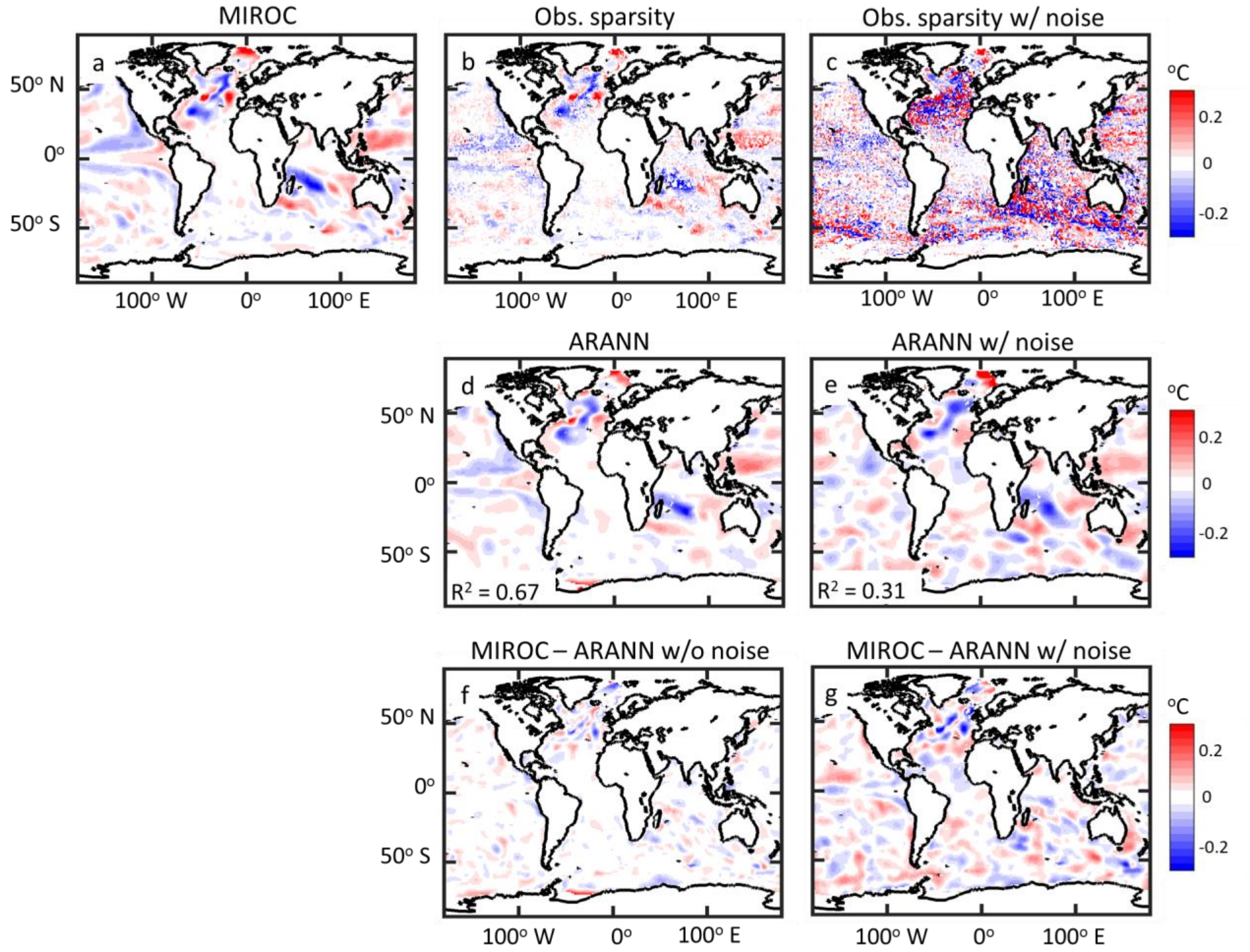


Temperature anomalies for Jan-Jun 2010 over the 900-1100 m depth interval for (a) a MIROC CMIP6 model run, (b) the MIROC model after being reduced to observed sparsity, and (c) the MIROC model at observed sparsity with added geophysical noise. The middle row shows (d) a single realization of the ARANN interpolation of the MIROC temperature anomalies and (e) the ARANN interpolation of the MIROC temperature anomalies with added geophysical noise. The bottom row shows (f) residuals between the original MIROC model and the ARANN interpolation and (g) residuals using an ARANN interpolation with additional geophysical noise added to the modeled temperature anomalies. The middle panels also show the $R^2$ between ARANN-reconstructed temperature anomalies (shown in each respective panel) and the original MIROC temperature anomalies in panel (a).

**Supplementary Fig. 12. Root-mean squared error of model reconstructions**

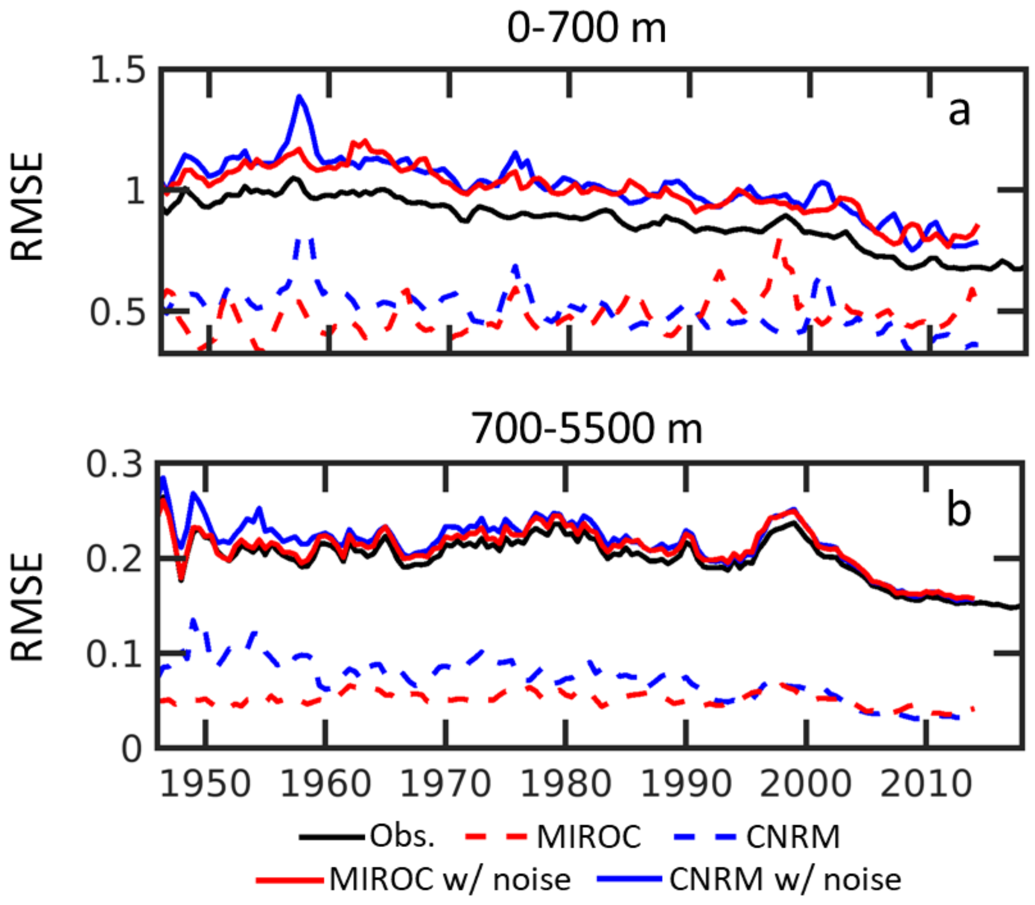


Root-mean squared error (RMSE) between the observed temperature anomalies and the ARANN reconstruction over time (black curve) for (a) the upper 700 m and (b) for the depth range 700-5500 m. Also shown for comparison is the RMSE between the CMIP6 modeled temperature anomalies and the ARANN reconstructions of the CMIP6 models for the MIROC (red dashed) and CNRM (blue dashed) models. Note the much larger RMSE for the ARANN reconstruction of the observed temperature anomalies than for the ARANN reconstruction of the modeled temperature anomalies. When additional noise is added to the modeled temperature anomalies to mimic geophysical noise in the observations, the RMSE between the MIROC (solid red) and CNRM (solid blue) temperature anomalies and the ARANN reconstruction is very similar to the RMSE between the ARANN and the observations (black).

**Supplementary Fig. 13. Impact of choice of instrument bias correction on ocean heat content estimates**

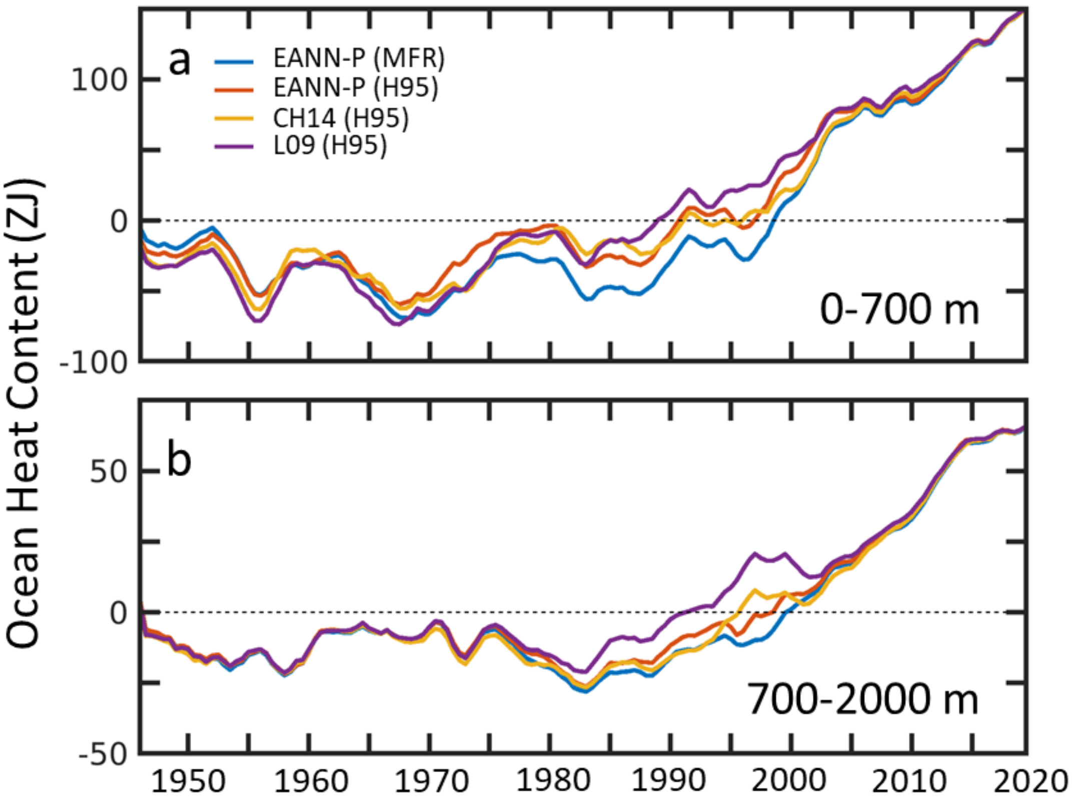


Global ocean heat content estimates for the depth intervals (a) 0-700 m and (b) 700-2000 m using various corrections for the systematic biases in the bathythermograph data, with their respective XBT fall rate equation in parentheses. Each curve represents the ensemble mean for a given correction method after running an ensemble of ARANNs comprised of 60 members. The XBT corrections used in this study are from L09[21], CH14[22,23], and EANN-P[24]. Two EANN-P calibrations are applied to the XBT data using either the original manufacturer fall rate equation (MFR) or a modified one (H95)[66].

250 **Supplementary Fig. 14. Impact of climatological choice on ocean heat content estimates**

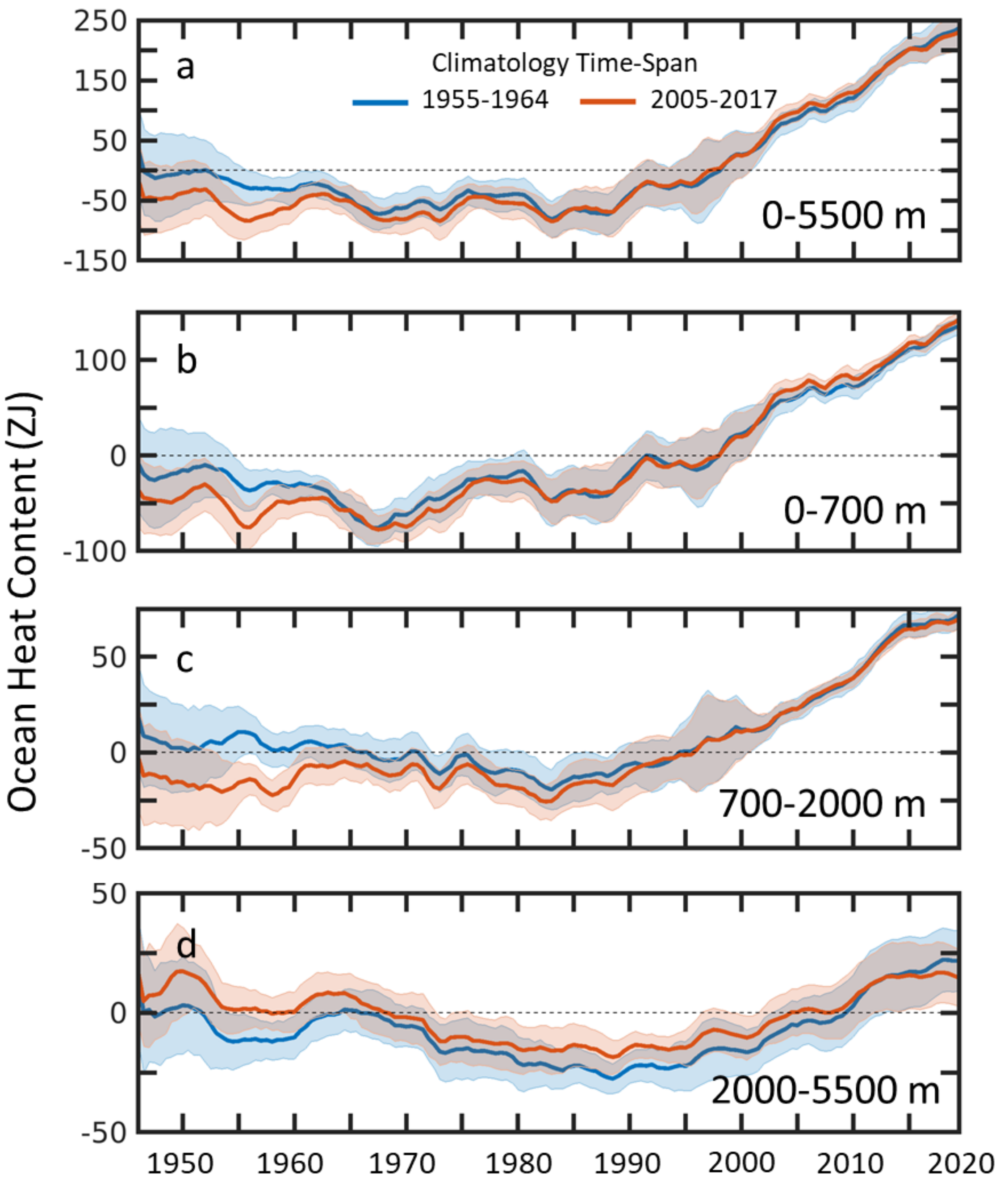


Global ocean heat content (OHC) reconstructions using two different climatologies to form the temperature anomalies before interpolation with the ARANN method. The first climatology uses only data from 2005-2017 (red curve), and the second climatology only uses data from 1955-
255 1964 (blue curve). Thick lines are the ensemble mean, and shading represents two standard deviations across 40 ARANN members using the four bathythermograph corrections shown in Supplementary Fig. 13. Four more decadal climatologies are combined with the ones shown here to produce the OHC estimates and uncertainties in the main text.

**Supplementary Fig. 15. Impact of warming rates in the upper and deep ocean on global ocean heat content**

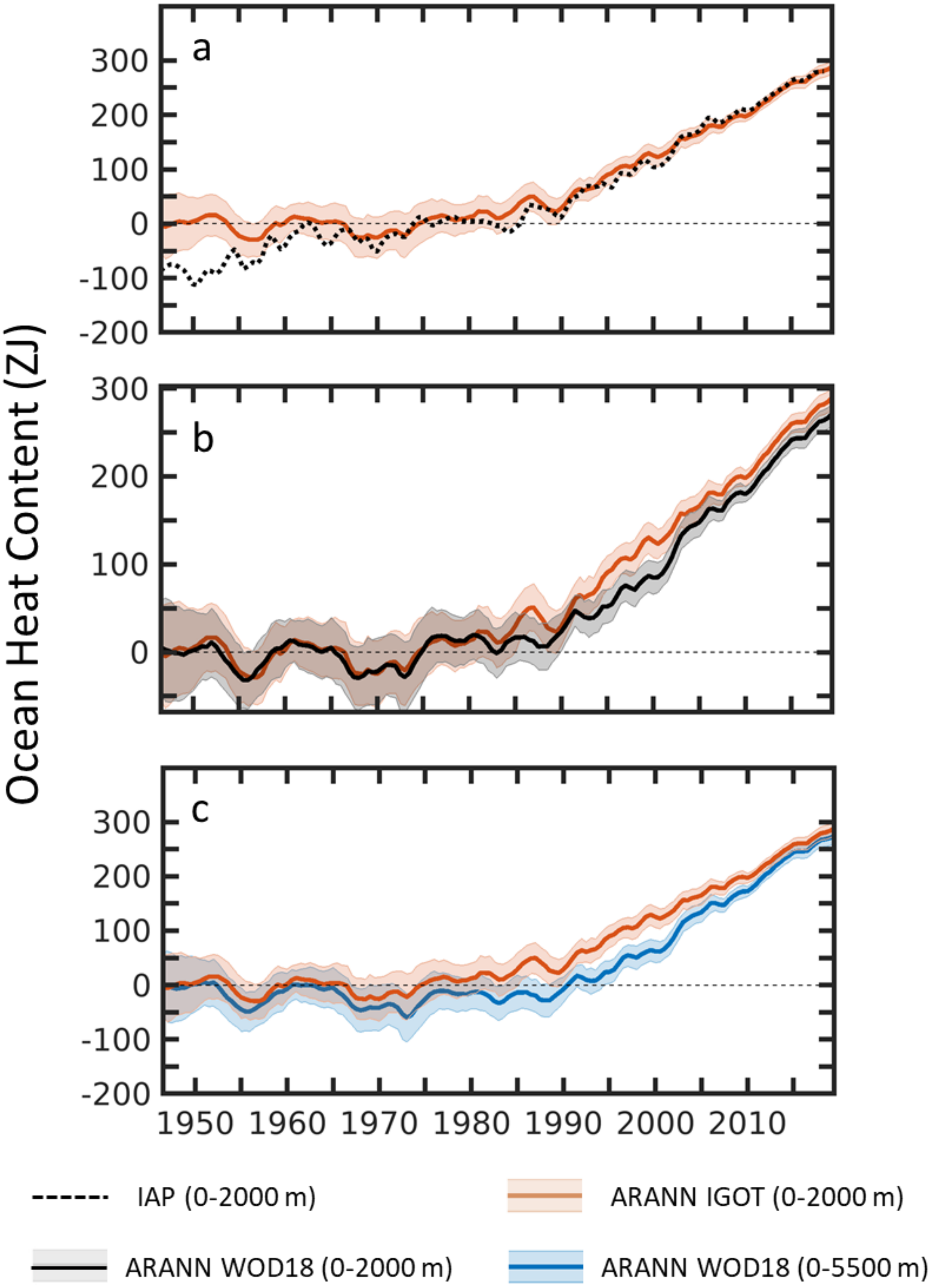


Global ocean heat content (OHC) estimates comparing the influence of the 0-2000 m OHC ARANN estimate versus the inclusion of the deep ocean below 2000 m. The official IAP[11] OHC estimate (dashed black) is compared to the 0-2000 m ARANN estimate that utilized the same IGOT dataset employed by the IAP (red), demonstrating differences solely due to methodology employed by the IAP and ARANN estimates (a). The 0-2000 m ARANN estimate using the IGOT dataset (red) is also compared to the estimate from the main text using the WOD18[18]

dataset and the CH14[10,11] instrumental bias correction (black) (b). Finally, the 0-2000 m ARANN estimate using the IGOT dataset (red) is compared to the 0-5500 m ARANN estimate using WOD18 dataset and the CH14 instrumental bias correction (blue) (c). The blue curve here is almost identical to that shown in Figure 1a, expect that only the CH14 bias correction is used. Anomalies for the ARANN estimates are set zero for year 1946. The IAP estimate is adjusted to the mean anomaly of the ARANN IGOT estimate for 2005-2017. Error bars represent 2 standard deviations across 20 ensemble members.
